# The emerging role of pyroptosis-related inflammasome pathway in atherosclerosis

**DOI:** 10.1186/s10020-022-00594-2

**Published:** 2022-12-21

**Authors:** Xiao-Dan Xu, Jia-Xian Chen, Lin Zhu, Shu-Ting Xu, Jian Jiang, Kun Ren

**Affiliations:** 1 Department of Pathology, The First Affiliated Hospital of Anhui Medical University, Hefei, 230022 Anhui People’s Republic of China; 2 Department of Cardiology, The Second Affiliated Hospital of Hainan Medical University, Haikou, 570100 Hainan People’s Republic of China; 3 College of Nursing, Anhui University of Chinese Medicine, Hefei, 230012 Anhui People’s Republic of China; 4 Department of Nephrology, The Affiliated Hospital of Dalian Medical University, Dalian, 116044 Liaoning People’s Republic of China; 5 Department of Organ Transplantation, The Second Affiliated Hospital of Hainan Medical University, Haikou, 570100 Hainan People’s Republic of China; 6 Institute of Clinical Medicine, The Second Affiliated Hospital of Hainan Medical University, Haikou, 570100 Hainan People’s Republic of China

**Keywords:** Atherosclerosis, Pyroptosis, Inflammation, Vascular endothelial cell, Macrophage, Vascular smooth muscle cell

## Abstract

Atherosclerosis (AS), a chronic sterile inflammatory disorder, is one of the leading causes of mortality worldwide. The dysfunction and unnatural death of plaque cells, including vascular endothelial cells (VEC), macrophages, and vascular smooth muscle cells (VSMC), are crucial factors in the progression of AS. Pyroptosis was described as a form of cell death at least two decades ago. It is featured by plasma membrane swelling and rupture, cell lysis, and consequent robust release of cytosolic contents and pro-inflammatory mediators, including interleukin-1β (IL-1β), IL-18, and high mobility group box 1 (HMGB1). Pyroptosis of plaque cells is commonly observed in the initiation and development of AS, and the levels of pyroptosis-related proteins are positively correlated with plaque instability, indicating the crucial contribution of pyroptosis to atherogenesis. Furthermore, studies have also identified some candidate anti-atherogenic agents targeting plaque cell pyroptosis. Herein, we summarize the research progress in understating (1) the discovery and definition of pyroptosis; (2) the characterization and molecular mechanisms of pyroptosis; (3) the regulatory mechanisms of pyroptosis in VEC, macrophage, and VSMC, as well as their potential role in AS progression, aimed at providing therapeutic targets for the prevention and treatment of AS.

## Introduction

Cardiovascular disease (CVD) refers to heart and blood vessel disorders, including heart failure, arrhythmia, hypertension, stroke, and myocardial infarction. In recent decades, CVD has become the primary reason for morbidity and mortality worldwide, irrespective of gender, ethnicity, and race. In China, approximately 300 million patients suffer from CVD (Du et al. 2019). Many risk factors, such as age, smoking, unhealthy diet, and dyslipidemia, have been identified to contribute to the incidence of CVD, the dominant cause of which is atherosclerosis (AS). AS, a metabolic and chronic inflammatory disease, is regarded as the pathological foundation of many CVDs. It is characterized by the deposition of lipid-rich foam cells and fibrous tissues within the intima of elastic arteries, resulting in the hardening and thickening of vascular walls and thrombus formation (Björkegren and Lusis 2022). The development of AS is accompanied by abnormal programmed cell death of plaque cells, e.g., vascular endothelial cell (VEC), macrophage and vascular smooth muscle cell (VSMC), and other types of vascular cells, leading to escalating inflammation. Specifically, in the early stage of AS, VEC*'*s injury, activation, and death caused by oxidized low-density lipoprotein (ox-LDL) can recruit monocytes and other circulating leukocytes for transendothelial migration. These monocytes in the endothelium can differentiate into macrophages and become foam cells by phagocytosing excess lipids. Besides, dying VEC can also promote the proliferation and migration of adjacent VSMC into the arterial intima, which increases the synthesis of extracellular matrix components, causing the formation of plaques and vasodilator dysfunction (Garcia and Blesso 2021). Although macrophage death effectively suppresses inflammation in incipient AS, it dramatically contributes to AS progression in advanced atherosclerotic lesions (Moore and Tabas 2011; Shibata and Glass 2009). Ineffective clearance of dying macrophages within vessel walls promotes the release of intracellular pro-inflammatory cytokines and lipids into extracellular space, resulting in necrotic core formation and plaque instability (Moore and Tabas 2011; Shibata and Glass 2009; Hansson 2005). VSMC death can reduce lesion cellularity, weaken the integrity of the fibrous cap, and accelerate plaque rupture (Clarke et al. 2010). These studies suggest that plaque cell death and inflammation are the two critical factors for AS progression.

Earlier, many researchers put forward that apoptosis and inefficient efferocytosis (clearance of apoptotic cells) are the main reason for inflammation in lesion cells (Tabas 2010; Vandivier et al. 2006). However, this opinion does not hold because cell lysis, not apoptosis, is widely observed in dying cells of human AS plaques (Ball et al. 1995; Naghavi et al. 2003), and the critical executor of apoptosis, caspase-3, is hardly detected in advanced atherosclerotic lesions (Kolodgie et al. 2000). Instead, there is an abundant expression of caspase-1 in both animal and human atherosclerotic plaques, and different research groups reported a strong positive correlation between caspase-1 and plaque instability (Ball et al. 1995; Kolodgie et al. 2000; Rossi et al. 2004). Caspases are a highly conserved family of protease enzymes that cleave their targeted proteins after aspartic acid residues. Through their specific cysteine protease activity, caspases play essential roles in facilitating programmed cell death (Man and Kanneganti 2016). Under physiological conditions, caspases are present in cell cytosol in the form of enzymatically inactive zymogens called pro-caspases. Under pathological conditions such as acute or chronic infection, pro-caspases can be cleaved to produce active caspases (Ramirez and Salvesen 2018). Generally, mammalian caspases comprise two categories: apoptotic and inflammatory. Based on the order of function in apoptotic execution, apoptotic caspases can be further subgrouped as initiator caspases (caspase-2, -8, -9, and -10), which act as proteolytic signal amplifiers and effector caspases (caspase-3, -6, and -7), which cleave cellular proteins proteolytically at their target sites to give rise to apoptosis (Galluzzi et al. 2016). The inflammatory caspase family involves caspase-1, -4, -5, -11, and -12, which have become crucial mediators of inflammation and immune response (Yin et al. 2013). Over 70 proteins have been identified as substrates for caspase-1, most of which are related to plaque inflammation and atherogenesis. For instance, the cleavage of pro-interleukin-1β (pro-IL-1β) and pro-IL-18 into mature IL-1β and IL-18 requires caspase-1 activation (Shen et al. 2010). Pyroptosis was described as a form of cell death at least two decades ago (Cookson and Brennan 2001; Brennan and Cookson 2000; Knodler et al. 2014). Distinct from apoptosis and necrosis, pyroptosis is closely related to the inflammatory response. It is characterized by cell swelling and lysis, nuclear condensation, DNA laddering, pore formation, and plasma membrane rupture, leading to rapid and massive leakage of intracellular contents and pro-inflammatory factors into extracellular space (Wei et al. 2022). Whitman et al. found that compared to apolipoprotein E null (apoE^−/−^) mice, the caspase-1 deficient (caspase-1^−/−^) apoE^−/−^ mice showed a 35% ~ 45% decrease in atherosclerotic lesion size in the ascending aorta. Furthermore, the expression of MHC class II (CD3), IL-1β, IL-18, and interferon-γ (IFN-γ) in the atherosclerotic plaques was reduced by 40–50% in caspase-1^−/−^apoE^−/−^ mice (Gage et al. 2012). Takahashi et al. observed similar phenomena in their studies (Usui et al. 2012). These findings indicate that caspase-1 and pyroptosis play critical roles in plaque cell death, inflammation, and AS progression.

## Discovery and definition of pyroptosis

The discovery and definition of pyroptosis have undergone several stages. Friedlander reported the earliest pyroptotic cell death in anthrax lethal toxin-treated macrophages in 1986 (Friedlander 1986). Later, Zychlinsky et al. observed that in *Shigella flexneri*-infected macrophages, the lytic cell death is not mediated by apoptosis executor caspase-3 but relies on the activation of caspase-1. Moreover, *Shigella flexneri* can kill macrophages from caspases-3, -11, and p53 knockout mice, and those macrophages overexpressing Bcl-2. In contrast, pharmacological inhibition or genetic ablation of caspase-1 significantly improved the resistance of macrophages to *Shigella flexneri* infection (Hilbi et al. 1997; Hilbi et al. 1998; Chen et al. 1996). Likewise, *Salmonella typhimurium*-induced macrophage death is also uniquely dependent on caspase-1 (Brennan and Cookson 2000; Hersh et al. 1999; Lara-Tejero et al. 2006). To distinguish it from apoptosis and necrosis, Cookson et al. defined this caspase-1-mediated pro-inflammatory cell death as "pyroptosis" ("pyro" meaning fire and "ptosis" meaning fall in Greek) in 2001 (Cookson and Brennan 2001). Subsequently, Kayagaki et al. found that in *Escherichia coli*-infected macrophages, caspase‐11, but not caspase-1, is required for cell pyroptosis. They proposed it as a non-canonical inflammasome pathway of pyroptosis (Kayagaki et al. 2011). Meanwhile, pyroptosis occurs in diverse non-monocytic cell types, including epithelial cells and keratinocytes (Knodler et al. 2014). Therefore, pyroptosis was redefined as caspase-1 or caspase-11(its human homologs caspase‐4/5)-dependent pro-inflammatory cell death (Shalini et al. 2015). In 2015, two independent research groups identified the gasdermin D (GSDMD) protein as the critical downstream target of caspase-1/11 (Shi et al. 2015a; Kayagaki et al. 2015). Moreover, caspase-3, caspase-8, and GSDME were also involved in the process of pyroptosis (Orning et al. 2018; Wang et al. 2017). Thus, pyroptosis was then redefined as "a category of programmed cell death that depends on gasdermins-induced plasma membrane pore formation, mainly (but not always) as a result of inflammatory caspases activation" by the Nomenclature Committee on Cell Death (NCCD) in 2018 (Galluzzi et al. 2018).

## Molecular mechanisms of pyroptosis

Pyroptosis is predominantly induced by the activation of pattern recognition receptors (PRRs). They are capable of recognizing the pathogen‐associated molecular patterns (PAMPs) and damage‐associated molecular patterns (DAMPs), including bacterial flagellum, LPS, ATP, double‐stranded DNA (dsDNA), and altered cellular components (Xia and Hollingsworth LRt et al.. 2020). Chemotherapy drugs and inflammatory cytokines, such as tumor necrosis factor (TNF-α) and interferon-γ (IFN-γ), are also stimuli for pyroptosis (Wang et al. 2017; Hou et al. 2020; Karki et al. 2021). Based on recognizing different triggers and activating distinct caspases, Kayagaki et al. classified pyroptosis pathways into two types: caspase-1-mediated canonical inflammasome pathway and caspase-4/5/11-mediated non-canonical inflammasome pathway (Kayagaki et al. 2011).

### Canonical inflammasome pathway

The caspase-1-mediated canonical inflammasome pathway of pyroptosis represents a critical mechanism in the innate immune system (Li et al. 2021a). Canonical inflammasomes consist of members of the nucleotide oligomerization domain (NOD)-like receptor (NLR) family, absent in melanoma 2 (AIM2) and pyrin proteins. NLR family is commonly composed of three parts: a central nucleotide-binding and oligomerization (NACHT) domain; the C-terminal leucine-rich repeats (LRR) domain; and the N-terminal caspase recruitment domain (CARD) or pyrin (PYD) domain, which is responsible for the recruitment of the adapter protein ASC (apoptosis-associated speck-like protein containing a CARD) (Zhen and Zhang 2019). AIM2 is a non-NLR inflammasome that comprises a PYD-signaling domain and a DNA-binding HIN-200 domain. (Lugrin and Martinon 2018). A comprehensive range of agonists, such as ATP, crystalline compounds, microbial pathogens, potassium (K^+^) efflux, and calcium (Ca^2+^) influx, can trigger the assembly of NLRP3 inflammasome (Kesavardhana et al. 2020; Sharma and Kanneganti 2021). In this process, lysosomal disruption and cathepsin release are crucial for NLRP3 activation (Orlowski et al. 2015). The catalytic activity of NIMA-related kinase-7 (NEK7) is also dispensable for activating the NLRP3 inflammasome (He et al. 2016). The NLRP1 inflammasome recognizes *bacillus anthracis* lethal toxin, *Shigella flexneri*, *Listeria monocytogenes*, and muramyl dipeptide (Mitchell et al. 2019). The NAIP/NLRC4 inflammasome responds to cytosolic Type III secretion system components and bacterial flagella (Nozaki et al. 2022). The AIM2 inflammasome specifically recognizes dsDNA (Lugrin and Martinon 2018). *Yersinia pestis-*induced Ras homologous A (RhoA) suppression can stimulate the pyrin inflammasome (Malik and Bliska 2020). After sensing the sign of infection or immunological challenge by PRRs, NLRs, AIM2, or pyrin can bind to ASC with signaling domains. The bound ASC then recruits the precursor of caspase-1 (pro-caspase-1) and induces the activation of caspase-1 (Aachoui et al. 2013). Interestingly, NLRC4 can directly recruit pro-caspase-1 and produce activated caspase-1 without binding to ASC (Case and Roy 2011). The activated caspase-1, in turn, cleaves pro-IL-1β and pro-IL-18 into mature IL-1β and IL-18. Meanwhile, activated caspases can also cleave full-length GSDMD (GSDMD-FL) protein into N-terminal fragments of GSDMD (GSDMD‐NT), which exerts pore-forming function and leads to pyroptotic cell lysis (Song et al. 2022; Sun et al. 2022). Most recently, Kayagaki et al. found that end‐stage plasma membrane rupture was not a passive osmotic lysis event but was dependent on ninjurin‐1 oligomerization (Kayagaki et al. 2021). Further studies are still needed to clarify the details of the process of the canonical inflammasome pathway to pyroptosis.

### Non-canonical inflammasome pathway

Kayagaki et al. first proposed the non-canonical inflammasome pathway of pyroptosis in 2011 (Kayagaki et al. 2011). The critical components of the non-canonical inflammasomes are caspase‐11 (murine) and caspase‐4/5 (human) rather than PRR proteins and adaptor proteins. The recognition of lipid A moiety of LPS in the cell wall of Gram-negative bacteria results in caspase‐11 activation. Activated caspase‐11 cleaves the downstream effector GSDMD-FL into GSDMD-NT, causing pore formation and secretion of IL-1α and high mobility group box 1 (HMGB1). Instead of directly maturing pro-IL-1β, caspase‐11-induced pyroptosis can indirectly process pro-IL-1β and promote the secretion of IL-1β by activating the NLRP3/ASC/caspase-1 pathway. The possible reasons may come from the following two aspects: (Du et al. 2019) pore formation by GSDMD-NT induces K^+^ efflux and activates the NLRP3 inflammasome ((Rühl and Broz 2015)); (2) activated caspase‐11 cleaves the pannexin‐1 channel and releases ATP, which in turn stimulates the P2X purinoreceptor 7 (P2X7) signaling, eventually inducing K^+^ efflux and NLRP3 activation (Yang et al. 2015). In human cells, caspase-4/5 performs the same function as murine caspase-11 (Martinon and Tschopp 2004). These findings suggest that the non-canonical inflammasomes are crucial regulators for the caspase-1-mediated canonical pathway to pyroptosis. More studies are necessary to unravel the mechanisms of non-canonical inflammasome-mediated pyroptosis in humans.

### The novel pathway to pyroptosis

**Fig. 1:**
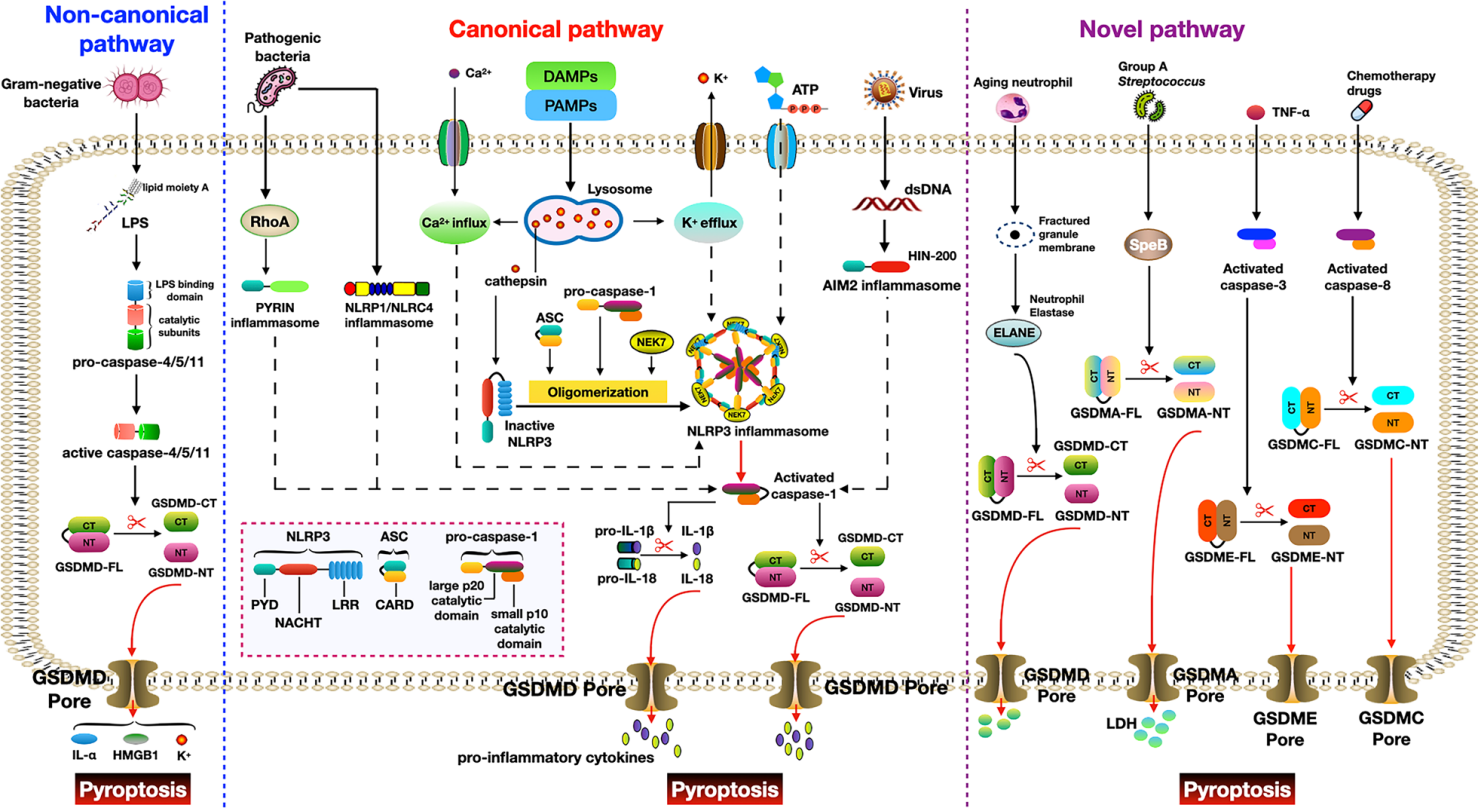
Molecular mechanisms of pyroptosis. Pyroptosis is a newly identified pro-inflammatory form of programmed cell death. According to the activation of distinct caspases and execution proteins, pyroptosis can be divided into three types: canonical pathway-mediated pyroptosis, non-canonical pathway-mediated pyroptosis, and novel pathway-induced pyroptosis. DAMPs, damage‐associated molecular patterns; PAMPs, pathogen‐associated molecular patterns; NLRP1/3, nucleotide‐binding domain, leucine-rich containing family, pyrin domain‐containing 1/3; NLRC4, NLR family CARD domain-containing 4; AIM2, absent in melanoma 2; NACHT, nucleotide-binding, and oligomerization; LRR, leucine-rich repeats; CARD, caspase recruitment domain; PYD, pyrin; ASC, apoptosis-associated speck-like protein containing a CARD; GSDMD-FL, full-length GSDMD; GSDMD-CT, C-terminal GSDMD; GSDMD-NT, N-terminal GSDMD; GSDMA-FL, full-length GSDMA; GSDMA-CT, C-terminal GSDMA; GSDMA-NT, N-terminal GSDMA; GSDME-FL, full-length GSDME; GSDME-CT, C-terminal GSDME; GSDME-NT, N-terminal GSDME; RhoA, Ras homologous A; SpeB, streptococcus exotoxin B; ELANE, neutrophil elastase; HMGB1, high mobility group box 1

GSDMD mainly contains two crucial domains: GSDMD-NT (≈30 KDa) and C-terminal fragments of GSDMD (GSDMD-CT) (≈20 KDa). GSDMD-NT is the pore-forming fragment, while GSDMD-CT acts as the inhibitory binding domain of GSDMD-NT. Different stimuli-induced activation of caspase-1/4/5/11 can cleave the GSDMD-FL at this linker region, which produces the activated GSDMD-NT and perforation on the membrane (Song et al. 2022; Sun et al. 2022). In addition to GSDMD, the gasdermin family proteins also include GSDMA, GSDMB, GSDMC, GSDME (DFNA5), and DFNB59 in humans. In aging neutrophils, GSDMD was cleaved and activated by the neutrophil-specific serine protease elastase (ELANE), which induces lytic cell death. Of note, this process is caspase-independent (Kambara et al. 2018). Deng et al. reported that infection of human A431 cells with group A *Streptococcus* (GAS) caused extensive pyroptosis with ballooning morphology of dying cells and massive lactate dehydrogenase (LDH) release. Mechanistically, intracellular GSDMA acts as a sensor and substrate of GAS cysteine protease SpeB. SpeB directly and selectively cleaves full-length GSDMA (GSDMA-FL) in the linker region after Gln246, producing N-terminal p27 fragments (GSDMA-NT). GSDMA-NT then promotes pore formation and gives rise to pyroptosis. Cysteine protease inhibitors can entirely abrogate SpeB-triggered pyroptotic cell death, but not the caspase inhibitors (Deng et al. 2022). Besides, Hou et al. found that in response to stimulators such as chemotherapy drugs and TNF‐α, apoptotic caspases (such as caspase‐3 and ‐8) induce pyroptotic cell death by cleaving gasdermin proteins (GSDMC, GSDMD, and GSDME). This process is not related to caspase-1/4/5/11 (Hou et al. 2020). These findings indicate that gasdermins can directly respond to certain stimuli and induce pyroptosis without the help of canonical or non-canonical inflammasomes, which we designated as the novel pyroptosis pathway (Fig. 1).

## Correlation between pyroptosis and AS

As is well known, AS is a chronic inflammatory disease. The pathological process of AS is driven by an imbalance between pro-inflammatory and pro-resolving mediators produced by VEC, macrophage, and VSMC in plaques. This leads to defective resolution of inflammation within the vessel walls (Doran 2022). Numerous studies have disclosed that pyroptosis is the leading cause of atherosclerotic inflammation. Previously, Zheng et al. found that NLRP3 and caspase-1, two vital execution proteins of pyroptosis, are strongly expressed in the aorta of patients with coronary AS, hypertension, or diabetes. Besides, aortic NLRP3 and caspase-1 levels are positively associated with coronary stenosis and atherosclerotic risk factors, including total cholesterol (TC), ox-LDL, and lipoprotein (a) (Zheng et al. 2013, 2014). In addition to NLRP3 and caspase-1, the expression of other pyroptosis-related factors, such as ASC, IL-1β, and IL-18, was also robustly increased in human atherosclerotic plaques compared to normal arteries, and the up-regulated levels of NLRP3 inflammasomes and inflammatory cytokines are closely related to plaque vulnerability (Paramel Varghese, et al. 2016). In low-density lipoprotein (LDL) receptor-deficient (LDLR^−/−^) mice, targeted disruption of individual components of NLRP3 inflammasome (NLRP3^−/−^, ASC^−/−^ or caspase-1^−/−^) resulted in a remarkable decrease in atherosclerotic lesion size. Compared to apoE^−/−^ mice, apoE^−/−^IL-1β^−/−^ mice displayed a 33% reduction in atheromatous plaques (Kirii et al. 2003). In the CANTOS (Canakinumab anti-inflammatory Thrombosis Outcomes Study) clinical trial, the patients treated with canakinumab, an antibody that neutralizes IL-1β, displayed a lower rate of recurrent CVD events than the patients receiving a placebo, irrespective of plasma lipid level (Ridker et al. 2017). These studies emphasized the correlation between pyroptosis and AS progression (Table 1).

**Table 1:** Factors that regulate pyroptosis of plaque cells in atherogenesis and the underlying mechanisms

Factors	Cell type	Py	Mechanisms/pathways	AS	Refs.
Ox-LDL	VEC	**↑**	ROS/NLRP3/caspase-1/GSDMD/IL-1β**↑**	**↑?**	Yin et al. (2015)
	MΦ	**↑**	CD36/ROS/NLRP3/caspase-1/GSDMD**↑**	**↑?**	Lin et al. (2013)
	MΦ	**↑**	IRF-1/ROS/NLRP3/caspase-1/GSDMD**↑**	**↑?**	Guo et al. (2019)
	MΦ	**↑**	NIX/mitophagy/ROS/NLRP3/caspase-1	**↑?**	Peng et al. (2020)
	MΦ	**↑**	Nrf2/HO-1/NQO-1/ROS/NLRP3/caspase-1	**↑?**	Qiu et al. (2022)
	MΦ	**↑**	p62/Nrf2/HO-1/caspase-1/GSDMD	**↑?**	Liu et al. (2021a)
	MΦ	**↑**	BRCC36/NLRP3/caspase-1/GSDMD	**↑?**	Singh et al. (2019)
	MΦ	**↑**	NF-κB/ABCA1/NLRP3/GSDMD	**↑?**	Li et al. (2022)
	MΦ	**↑**	caspase-11/GSDMD/IL-1β**↑**	**↑**	Jiang et al. (2021)
	VSMC	**↑**	NLRP3/caspase-1/GSDMD/IL-1β**↑**	**↑**	Puylaert et al. (2022)
	VSMC	**↑**	NF-κB/AIM2/caspase-1/GSDMD**↑**	**↑**	Pan et al. (2018)
SAL	VEC	**↓**	NLRP3/caspase-1/GSDMD/IL-1β**↓**	**↓**	Xing et al. (2020)
Nicotine	VEC	**↑**	ROS/NLRP3/caspase-1/GSDMD/IL-1β**↑**	**↑**	Wu et al. (2018)
	MΦ	**↑**	HDAC6/NF-κB/NLRP3/caspase-1/GSDMD	**↑**	Xu et al. (2021b)
TMAO	VEC	**↑**	SDHB/ROS/NLRP3/caspase-1/GSDMD**↑**	**↑**	Wu et al. (2020)
Melatonin	VEC	**↓**	Nrf2/HO-1/NQO-1/ROS/NLRP3/caspase-1	**↓**	Zhao et al. (2021)
	VEC	**↓**	MEG3/miR-223/NLRP3/caspase-1/GSDMD	**↓**	Zhang, et al. (2018)
	VEC	**↓**	TET2/UQCRC1/NLRP3/caspase-1/IL-1β	**↓?**	Zeng et al. (2021a)
	VEC	**↓**	RORα/miR-223/STAT3/caspase-1/GSDMD	**↓?**	Yi and Yang (2021)
FGF21	VEC	**↓**	ROS/NLRP3/caspase-1/GSDMD/IL-1β**↓**	**↓**	Zeng et al. (2020)
Cadmium	VEC	**↑**	ROS/NLRP3/caspase-1/GSDMD/IL-1β**↑**	**↑?**	Oliveira et al. (2019; Chen et al. 2016)
Hcy	VEC	**↑**	ROS/NLRP3/caspase-1/IL-1β**↑**	**↓?**	Ma et al. (2022), Xi et al. 2016)
LSS	VEC	**↑**	miR-181-5p/STAT3/NLRP3/caspase-1	**↑?**	Zhang et al. (2020), Xu et al. 2021a)
	VEC	**↑**	TET2/SDHB/ROS/NLRP3/caspase-1	**↑?**	Zhang et al. (2020), Chen et al. (2021)
Acrolein	VEC	**↑**	ROS/NLRP3/caspase-1/GSDMD**↑**	**↑?**	Srivastava et al. (2011), Jiang et al. (2018)
Estrogen	VEC	**↓**	ERα/autophagy/NLRP3/caspase-1/GSDMD	**↓**	Meng et al. (2021)
BDNF	VEC	**↓**	KLF2/HK1/NLRP3/caspase-1/IL-1β	**↓?**	Bi et al. (2020), Jin et al. (2021)
miR-30c-5p	VEC	**↓**	FOXO3/NLRP3/caspase-1/IL-1β**↓**	**↓?**	Vilahur (2017), Li et al. (2018)
Colchicine	VEC	**↓**	AMPK/SIRT1/ROS/NLRP3/caspase-1	**↓?**	Li et al. (2021b), Yang et al. (2020)
HDAC11	VEC	**↓**	ERG/NLRP3/caspase-1/GSDMD	**↓?**	Yao et al. (2022)
	VEC	**↓**	ERG/caspase-3/GSDME	**↓?**	Yao et al. (2022)
Sal B	EPC	**↓**	AMPK/FOXO4/KLF2/NLRP3/caspase-1 Syndecan-4/Rac1/ATF2/NLRP3/caspase-1	**↓?**	Tang et al. (2022)
	EPC	**↓**		**↓?**	Tang et al. (2022)
ApoM-S1P	VEC	**↓**	S1PR2/PI3K/AKT/NLRP3/caspase-1	**↓?**	Liu and Tie (2019)
Oxymatrine	VEC	**↓**	SIRT1/Nrf2/HO-1/ROS/NLRP3//caspase-1	**↓?**	Jin, et al. (2021)
CC	MΦ	**↑**	cathepsin/NLRP3/caspase-1/GSDMD**↑**	**↑?**	Duewell et al. (2010), Rajamäki et al. (2010)
BMSC-MV	MΦ	**↑**	miR-223/NLRP3/caspase-1/GSDMD	**↑**	Lin et al. (2021)
PM_2.5_	MΦ	**↑**	NLRP3/caspase-1/GSDMD/IL-1β**↑**	**↑**	Du et al. (2018)
Pg	MΦ	**↑**	CD36/TLR2/NLRP3/caspase-1/IL-1β**↑**	**↑**	Brown et al. (2015)
	VSMC	**↑**	PPP1CC/HMGB1/TLR9/AIM2/caspase-1	**↑?**	Liu et al. (2021b)
Sinapic acid	MΦ	**↓**	MALAT1/miR-23c/ELAVL1/NLRP3	**↓?**	Han et al. (2018)
dsDNA	MΦ	**↑**	AIM2/caspase-1/GSDMD/IL-1β**↑**	**↑**	Paulin et al. (2018)
Adiponectin	VSMC	**↓**	miR-133a/NLRP3/caspase-1/GSDMD/IL-β	**↓?**	Duan et al. (2020)

Py: pyroptosis; VEC: vascular endothelial cell; MΦ: macrophage; VSMC: vascular smooth muscle cell; AS: atherosclerosis; ROS: reactive oxygen species; NLRP3: nucleotide‐binding domain, leucine-rich containing family, pyrin domain‐containing 3; CD36: cluster of differentiation 36; IRF-1: interferon regulatory factor 1; NIX: NIP3-like protein X; Nrf2: nuclear factor erythroid 2‑related factor 2; HO-1: heme oxygenase‑1; NQO-1: NADPH quinone oxido‑reductase‑1; AIM2: absent in melanoma 2; SAL: salidroside; HDAC: histone deacetylase; TMAO: trimethylamine *N*‐oxide; SDHB: succinate dehydrogenase complex subunit B; MEG3: maternally expressed 3; TET2: ten-eleven translocation 2; UQCRC1: ubiquinol-cytochrome c reductase core protein 1; RORα: retinoid-related orphan receptor α; STAT3: signal transducer and activator of transcription 3; FGF21: fibroblast growth factor 21; Hcy: homocysteine; LSS: low shear stress; Erα: estrogen receptor α; BDNF: brain-derived neurotrophic factor; KLF2: kruppel-like family of transcription factor 2; HK1: hexokinase 1; FOXO3: forkhead box O3; SIRT1: sirtuin1; ERG: erythroblast transformation specific-related gene; GSDME: gasdermin E; Sal B: salvianolic acid B; EPC: endothelial progenitor cell; ATF2: activating transcription factor-2; apoM-S1P: apolipoprotein M and sphingosine-1-phosphate complex; S1PR2: S1P receptor 2; PI3K: phosphoinositide 3-kinase; AKT: protein kinase B; CC: cholesterol crystal; BMSC-MV: bone marrow-derived mesenchymal stem cell macrovesicle; Pg: *Porphyromonas gingivalis*; TLR2: Toll-like receptor 2; PPP1CC: protein phosphatase 1 catalytic subunit gamma; HMGB1: high mobility group box 1; ELAVL1: embryonic lethal abnormal vision-like 1; dsDNA: double‐stranded DNA. **↓**: suppress; **↑**: promote; **?**: probably (not verified)

### VEC pyroptosis—a vital contributor to the initiation of AS

The vascular endothelium forms a continuous monolayer on the inner surface of the vessel wall. VEC maintains the integrity of the vessel walls and keeps blood leukocytes and lymphocytes quiescent. Endothelial activation is the first and essential step for AS progression. It increases the expression of adhesion molecules to make VEC more adhesive. Besides, it up-regulates the secretion of inflammatory cytokines and chemokines to attract monocytes for transendothelial recruitment (Garcia and Blesso 2021). Yin et al. identified that caspase-1 in VEC can sense early hyperlipidemia in mice fed an HFD for 3 weeks. Compared to apoE^−/−^ mice, caspase-1^−/−^apoE^−/−^ mice showed a robust decrease in endothelial activation, vascular inflammation, and monocyte recruitment, leading to the minor atherosclerotic lesion in the early stage of AS. In human aortic endothelial cells (HAECs), ox-LDL treatment significantly induced pyroptosis, as evidenced by disruption of plasma membrane integrity and increased NLRP3, caspase-1, and IL-1β transcripts (Yin et al. 2015). Inhibition of VEC pyroptosis by salidroside (SAL) can markedly decrease lipid deposition and plaque formation in apoE^−/−^ mice (Xing et al. 2020). These findings demonstrate the pivotal role of VEC pyroptosis in the starting stage of AS.

Multiple pathological factors aggravated AS by stimulating VEC pyroptosis via the reactive oxygen species (ROS)/NLRP3/caspase-1 pathway. Nicotine-treated apoE^−/−^ mice showed larger atherosclerotic plaques and elevated serum concentrations of IL-1β and IL-18, while NLRP3 knockdown in vivo significantly compromised these effects. The LDH release and expression of NLRP3, ASC, caspase-1, IL-1β, and IL-18 were increased considerably in aortic VECs; in contrast, adding N-acetylcysteine (NAC), a ROS inhibitor, reversed these phenomena (Wu et al. 2018). Administration of trimethylamine N‐oxide (TMAO) into apoE^−/−^ mice caused a robust increase in atherosclerotic lesion size, serum IL‐1β levels, lipid accumulation, and collagen content in the aortic root. TMAO also increased succinate dehydrogenase complex subunit B (SDHB), NLRP3, and caspase‐1 expression in VECs of the lesion area. In vitro*,* incubating human umbilical vascular endothelial cells (HUVECs) with TMAO induced mitochondrial damage, reduced ATP production, and increased expression of SDHB and ROS, leading to activation of NLRP3/caspase-1 pathway, eventually pyroptosis (Wu et al. 2020). Zhao et al. found that melatonin treatment reduced carotid artery intimal hyperplasia in atherosclerotic rats. Mechanistically, melatonin activated nuclear factor erythroid 2‑related factor 2 (Nrf2) / heme oxygenase‑1 (HO-1) pathway and decreased the levels of ROS and pyroptosis-related proteins (NLRP3, caspase-1, IL‐1β, IL‐18) in aortic VECs. HAECs incubated with melatonin showed similar results, indicating that melatonin inhibits AS progression by suppressing VEC pyroptosis through Nrf2/HO-1/ROS/NLRP3 pathway (Zhao et al. 2021). Moreover, the MEG3/miR-223/NLRP3 axis, TET2/ UQCRC1/NLRP3 (ten-eleven translocation 2/ubiquinol-cytochrome c reductase core protein 1/NLRP3) axis, and RORα/miR-223/STAT3 (nuclear receptor retinoid-related orphan receptor α/miR-223/signal transducer and activator of transcription 3) axis are also involved in melatonin-induced repression of VEC pyroptosis (Zhang, et al. 2018; Zeng et al. 2021a; Yi and Yang 2021). Zeng et al. found that intraperitoneal infection of apoE^−/−^ mice with recombinant fibroblast growth factor 21 (FGF21) resulted in significantly smaller atherosclerotic lesions and improved lipid profile. In ox-LDL-stimulated HUVECs, FGF21 suppressed mitochondrial division and decreased ROS production, leading to attenuated NLRP3 inflammasome activation and pyroptosis (Zeng et al. 2020). Chronic exposure of HFD-fed apoE^−/−^ mice to cadmium (Cd^2+^) enlarged atherosclerotic plaques, increased plasma cholesterol levels, and induced endothelial dysfunction (Oliveira et al. 2019). Further research unraveled that Cd^2+^ treatment amplified mitochondrial ROS (mtROS) generation, stimulated the NLRP3/caspase-1 pathway, and increased the expression of IL-6, IL-8, TNF-α, and monocyte chemoattractant protein 1(MCP-1), resulting in proptosis in HUVECs (Chen et al. 2016). Hyperhomocysteinemia (HHcy) is an independent risk factor for AS (Ma et al. 2022). Co-incubating HUVECs with HHcy augmented ROS production and stimulated NLRP3 inflammasome assembly, causing caspase-1 activation and subsequent pyroptosis (Xi et al. 2016). Low shear stress (LSS) contributes to the initiation and progression of AS by promoting endothelial activation and inflammation (Zhang et al. 2020). LSS can potently promote NLRP3 inflammasome-mediated VEC pyroptosis via the miR-181b-5p/STAT3/NLRP3 axis or TET2/SDHB/ROS pathway (Xu et al. 2021a; Chen et al. 2021). Dietary exposure to acrolein increased atherosclerotic lesion formation in the aorta of apoE^−/−^ mice (Srivastava et al. 2011). In vitro*,* acrolein accelerated mitochondrial damage, suppressed autophagy, and promoted NLRP3 inflammasome activation in a ROS-dependent manner, leading to HUVEC pyroptosis (Jiang et al. 2018).

Since VEC pyroptosis's role in AS attracts more attention, many researchers have focused on exploring the regulatory mechanisms beneath VEC pyroptosis. Treatment of apoE^−/−^ mice with estrogen significantly attenuated the plaque area in the aorta, decreased TC, TG, and LDL-C levels, and increased HDL-C levels in the plasma. Furthermore, estrogen suppressed VEC pyroptosis via activation of estrogen receptor α (ERα)-mediated autophagy in the cardiac aortas of apoE^−/−^ mice and in HUVECs (Meng et al. 2021). Brain-derived neurotrophic factor (BDNF) is a well-demonstrated anti-AS factor (Bi et al. 2020). It can attenuate ox-LDL-induced NLRP3 inflammasome assembly and pyroptosis in HUVECs by preserving mitochondrial homeostasis through the KLF2/HK1 (kruppel-like family of transcription factor 2/hexokinase 1) pathway (Jin et al. 2021). miR-30c-5p, a promising predictive biomarker for AS prevention, inhibited HAECs pyroptosis through the forkhead box O3 (FOXO3)/NLRP3 pathway (Vilahur 2017; Li et al. 2018). Colchicine, a potential anti-AS drug (Li et al. 2021b), inhibited cholesterol crystal-induced HUVEC pyroptosis and inflammation via the AMPK/Sirtuin1 (SIRT1)/ROS/NLRP3 pathway (Yang et al. 2020). Histone deacetylase (HDAC11) can form a complex with erythroblast transformation specific (ETS)-related gene (ERG) in TNF-α-treated HUVECs, which inhibits cell pyroptosis through NLRP3/caspase-1/GSDMD or caspase-3/GSDME pathway (Yao et al. 2022). Salvianolic Acid B (Sal B) can attenuate pyroptosis of bone marrow-derived endothelial progenitor cells (BM-EPCs) by suppressing endoplasmic reticulum (ER) stress and NLRP3 inflammasome activation via the AMPK/FOXO4/KLR2 and Syndecan-4/Rac1/ATF2 pathways (Tang et al. 2022). Apolipoprotein M and sphingosine-1-phosphate complex (apoM-S1P) can alleviate HUVEC pyroptosis by activating the S1PR2/PI3K/AKT signaling pathway (Liu and Tie 2019). Oxymatrine, a bioactive component from the Chinese herb *Sophora flavescens*, attenuated ox‑LDL-induced HUVEC pyroptosis and injury via the SIRT1/Nrf2/HO-1/ROS/NLRP3 signaling pathway (Jin et al. 2021). miR-30c-5p, colchicine, HDAC11, Sal B, apoM-S1P, and oxymatrine may delay AS progression due to their anti-pyroptotic and anti-inflammatory effects in VECs, although the direct evidence is missing.

### Macrophage pyroptosis—a critical player in the progression of AS

Disturbance of endothelial permeability and functional impairment causes the release of inflammatory cytokines, P-selectin, intracellular adhesion molecule-1 (ICAM-1), and vascular cell adhesion molecule-1 (VCAM-1), leading to the recruitment of monocytes into the subendothelium. Then, recruited monocytes internalize modified LDL, such as ox-LDL, and differentiate into macrophages and cholesterol-rich foam cells, which drives inflammatory responses during lesion formation (Tall et al. 2022). Hypercholesterolemic mice lacking differentiated macrophages displayed minimal AS plaques (Binder et al. 2002). In early AS, moderate macrophage death reduces metalloproteinase (MMP) synthesis and alleviates inflammatory response. In contrast, in the late stage of AS, excessive macrophage death and ineffective efferocytosis expand the necrotic lipid core, amplify inflammation and enhance plaque vulnerability (Susser and Rayner 2022). CD68-positive macrophages in advanced atherosclerotic lesions showed a predominant activation of the NLRP3 inflammasome signaling pathway (Paramel Varghese et al. 2016; Shi et al. 2015b). Compared to LDLR^−/−^ mice receiving NLRP3^+/+^ bone marrow, LDLR^−/−^ mice transplanted with NLRP3^−/−^ bone marrow displayed reduced circulating levels of IL-1β and IL-18 and smaller atherosclerotic lesion size (Duewell et al. 2010). These studies indicate that macrophage pyroptosis is vital for forming and rupturing atherosclerotic plaques. Cholesterol crystals and ox-LDL are two potent endogenous stimulators for macrophage pyroptosis during AS progression. The development of human atherosclerotic lesions from fatty streaks to advanced lesions is accompanied by a sharp increase in cholesterol crystals (Guyton and Klemp 1993). In high-cholesterol diet-fed apoE^−/−^ mice, the deposition of cholesterol crystals in necrotic cores and plaque macrophages increased steadily with diet feeding. Treatment of human peripheral blood mononuclear cells (PBMCs) with cholesterol crystals induced a robust, dose-responsive release of IL-1β in a caspase-1-dependent manner. In NLRP3-/ASC-deficient macrophages, cholesterol crystals failed to induce caspase-1 cleavage and IL-1β release. Further data showed that cholesterol crystals promoted NLRP3 inflammasome activation and IL-1β secretion in macrophages by increasing lysosomal rupture and efflux of cathepsin and K^+^ (Duewell et al. 2010; Rajamäki et al. 2010).

In addition to cholesterol crystals, ox-LDL can promote macrophage pyroptosis through many mechanisms. ROS is a well-known sensor for NLRP3. ROS generation significantly activates the NLRP3 inflammasome and caspase-1. Lin et al. reported that ox-LDL interacted with a cluster of differentiation 36 (CD36), increased ROS production, and induced NLRP3 inflammasome-mediated pyroptosis in human THP-1 macrophages (Lin et al. 2013). Inhibition of IFN regulatory factor 1 (IRF-1) can compromise pyroptosis in ox-LDL-treated macrophages by decreasing ROS production and subsequent activation of the NLRP3/caspase-1 pathway (Guo et al. 2019). NIP3-like protein X (NIX) is a pyroptosis-related protein located in the outer mitochondrial membrane. NIX-mediated mitochondrial autophagy (mitophagy) decreases ROS production and maintains mitochondrial integrity, inhibiting the activation of the NLRP3/caspase-1/IL-1β pathway. Silencing NIX amplifies ox-LDL-induced macrophage pyroptosis, indicating that ox-LDL propels macrophage pyroptosis by suppressing NIX-mediated mitophagy (Peng et al. 2020). Additionally, ox-LDL can inhibit Nrf2 nuclear translocation and decrease the expression of HO-1 and NADPH quinone oxide‑reductase‑1 (NQO-1) in human macrophages, leading to more ROS production, NLRP3 inflammasome activation, and eventually pyroptosis (Qiu et al. 2022). Interestingly, Liu et al. observed that ox-LDL treatment remarkably triggered p62 accumulation by impairing autophagy, causing over-activation of the Nrf2/HO-1 pathway and pyroptosis in THP-1-differentiated macrophages (Liu et al. 2021a). BRCC36 is a deubiquitinating enzyme. Singh et al. found that ox-LDL activated BRCC36 and promoted NLRP3/caspase-1-mediated pyroptosis in murine Raw 264.7 macrophages. Specifically, ox-LDL intensified the proteasomal and deubiquitinating activity of BRCC36, which decreased proteasomal degradation of the NLRP3 molecule and activated the NLRP3 inflammasome (Singh et al. 2019). Most recently, Li et al. found that inhibition of nuclear factor kappa-B (NF-κB) phosphorylation and enhancement of ATP binding cassette subfamily A1 (ABCA1)-mediated cholesterol efflux can alleviate pyroptosis in ox-LDL-stimulated macrophages (Li et al. 2022). Suppression of pyroptosis by VX-765, a specific caspase-1 inhibitor, increased cholesterol efflux and suppressed macrophage foam cell formation, suggesting the crosstalk between pyroptosis and cholesterol deposition through the NF-κB/ABCA1 pathway (Jin et al. 2022). In addition to caspase-1, ox-LDL can also induce caspase-4/11-GSDMD-mediated pyroptosis and subsequent inflammation in macrophages, which is involved in the pathogenesis of AS (Jiang et al. 2021).

Macrophage pyroptosis contributes significantly to AS progression. Treatment of apoE^−/−^ mice with MCC590, a selective inhibitor of NLRP3, markedly reduced the number of macrophages in the plaque and plasma IL-1β levels, resulting in a smaller carotid artery plaque size (Heijden et al. 2017). MCC950 inhibited the NLRP3/ASC/caspase-1/GSDMD signaling pathway and alleviated macrophage pyroptosis in the aorta of apoE^−/−^ mice (Zeng et al. 2021b). Besides, injection of high-fat diet (HFD)-fed apoE^−/−^ mice with bone marrow-derived mesenchymal stem cells microvesicles (BMSCs-MVs) significantly reduced macrophage numbers and mitigated lipid accumulation in AS-prone regions of the aortic root. BMSCs-MVs decreased the expression of pyroptosis-related proteins (NLRP3, caspase-1, GSDMD, IL-1β, and IL-18) both in the aorta and high glucose-stimulated bone marrow-derived macrophages (BMDMs). Moreover, BMSCs-MVs carrying miR-223 directly targeted NLRP3 3′UTR and down-regulated NLRP3 expression, which blocked NLRP3 inflammasome activation and pyroptosis in BMDMs (Lin et al. 2021). Nicotine, the practical component of tobacco, exerts pro-atherogenic effects in many ways (Fu et al. 2021). Nicotine-treated apoE^−/−^ mice showed larger aortic lesions and more lipid accumulation within plaques. Nicotine up-regulated caspase-1 expression in plaque macrophages and increased IL-1β and IL-18 levels in serum. Co-incubating RAW264.7 cells with nicotine increased HDAC6 expression and NF-κB p65 nuclear translocation. The binding of p65 to the NLRP3 promoter region augmented NLRP3 expression, triggering NLRP3 inflammasome activation and pyroptosis (Xu et al. 2021b). Exposure of HFD-fed apoE^−/−^ mice to PM_2.5_ aggravated atherosclerotic plaque formation, amplified NLRP3 inflammasome signaling in the aortic root, and elevated plasma cholesterol levels. Moreover, NLRP3 expression and caspase-1 activity were increased in macrophages isolated from PM_2.5_-treated apoE^−/−^ mice, indicating that macrophage pyroptosis is involved in PM_2.5_-induced atherogenesis (Du et al. 2018). Infection of murine macrophages with *Porphyromonas gingivalis* (Pg) induced activation of the NLRP3 inflammasome, IL-1β release, and pyroptosis via the CD36/toll-like receptor 2 (TLR2) pathway. In LDLR^−/−^ mice, Pg increased lesion burden and worsened AS by increasing CD36 (Brown et al. 2015). Chronic administration of sinapic acid (SA) into diabetic atherosclerotic (DA) rats decreased serum IL-1β levels and the expression of pyroptotic proteins (NRLP3, ASC, and caspase-1) in BMDMs. BMDMs from DA rats exhibited increased metastasis-associated lung adenocarcinoma transcript 1 (MALAT1), which sponged miR-23c and elevated the expression of embryonic lethal abnormal vision-like 1 (ELAVL1). ELAVL1 is an upstream activator of NLRP3 inflammasome. SA can suppress NLRP3 inflammasome activation and pyroptosis of BMDMs through inhibition of the MALAT1/miR-23c/ELAVL1 signaling (Han et al. 2018). The AIM2 inflammasome specifically recognizes cytosolic dsDNA, leading to the release of IL-1β and IL-18 and pyroptosis. In the early stages of AS, dsDNA was hardly detected, while in advanced atherosclerotic plaques, larger quantities of dsDNA and abundant AIM2 expression were observed. The expression of AIM2 was restricted to plaque macrophages, where dsDNA colocalized with AIM2. AIM2^−/−^apoE^−/−^ mice showed a significant decrease in the necrotic core area and production of IL-1β and IL-18 within plaques (Paulin et al. 2018). Fidler et al. unraveled that macrophage-specific mutation of the Janus kinase 2 (m-Jak2^−/−^) in LDLR^−/−^ mice remarkably increased serum IL-18 levels and macrophage proliferation within atheromas, causing larger lesion areas and prominent formation of necrotic cores. Deletion of caspase-1, -11 or the pyroptosis executioner GSDMD notably abrogated these adverse effects. Jak2 mutation increased the expression of AIM2 and oxidative DNA damage in BMDMs. Compared to m-Jak2^−/−^ mice, m-Jak2^−/−^AIM2^−/−^ mice had smaller atherosclerotic lesions and necrotic cores, whereas these phenomena were not detected in m-Jak2^−/−^NLRP3^−/−^ mice (Fidler et al. 2021). These findings suggest that in addition to NLRP3, AIM2 inflammasome-mediated macrophage pyroptosis aggravates AS.

### VSMC pyroptosis—a great promoter of AS plaque rupture

The migration of VSMCs from the media layer into the intima layer is an important event in atheroma formation. Intimal VSMCs secrete the extracellular matrix to form a fibrous cap that covers the plaque. In the advanced stage of AS, VSMC death triggers severe vascular inflammation and less collagen content, leading to thinner fibrous caps and plaque rupture (Grootaert et al. 2018). VSMC pyroptosis, but not apoptosis, induces persistent inflammation and destroys the blood vessel walls*'* structure (He et al. 2021; Chang et al. 2013). In human plaques, GSDMD-NT is mainly expressed in VSMC-rich areas of human plaques. VSMCs from human plaques exhibit increased expression of pyroptosis‐related proteins, including NLRP3 and ASC (Paramel Varghese, et al. 2016). Compared to apoE^−/−^GSDMD^+/+^ mice, apoE^−/−^GSDMD^−/−^ mice had smaller atherosclerotic plaques in the brachiocephalic artery. VSMCs from apoE^−/−^GSDMD^−/−^ mice are less susceptible to pyroptosis inducers (Puylaert, et al. 2022). Zhang et al. reported that in human carotid plaques and aortic roots of apoE^−/−^ mice, the location of caspase-1 and IL-1β overlapped with α-SMA-positive areas. Treatment of aortic VSMCs with ox-LDL significantly induced pyroptosis in an NLRP3 inflammasome-dependent manner. Administration of apoE^−/−^ mice with VX-765 significantly decreased the number of α-SMA and IL-1β co-stained cells in plaque and alleviated lipid deposition in the whole aorta, suggesting that VSMC pyroptosis exacerbates AS (Li et al. 2020). Plus, treatment of murine VSMCs with high ox-LDL markedly up-regulated AIM2 expression and pyroptosis through NF-κB signaling. AIM2 overexpression in apoE^−/−^ mice promoted VSMC pyroptosis, elevated ICAM-1 level in aortic roots, and developed larger atherosclerotic lesions (Pan et al. 2018). Adiponectin (APN) is a fatty tissue-specific protein with anti-inflammation and anti-AS functions. APN can attenuate VSMC pyroptosis by increasing miR-133a levels and inhibiting NLRP3 inflammasome activation, which improves aortic dissection in vivo (Duan et al. 2020). Pg is a validated pro-atherogenic bacterium. Pg can increase the proliferation and pyroptosis of VSMCs by activating the circRNA PPP1CC/HMGB1/TLR9/AIM2 pathway (Liu et al. 2021b). The underlying mechanisms of VSMC pyroptosis and their relationship with atherogenesis require more exploration.

## Conclusions and future perspectives

IN recent decades, the emerging role of pyroptosis in the initiation, progression, and complications of AS has craved more and more attention. Pyroptosis of VEC damages the endothelial function and promotes the release of pro-inflammatory cytokines, which stimulate the migration of monocytes to form early plaques (Libby et al. 2019). Pyroptosis of macrophages further releases cytokines and induces foam cell formation (Jin et al. 2022). Pyroptosis of VSMC reduces the size of the fibrous cap by decreasing collagen and matrix content, aggravating plaque instability and rupture. However, several limitations are apparent in the studies of pyroptosis and atherogenesis, presented as follows: (1) most experimental works focus on the pyroptosis of VEC and macrophages, whereas the role of VSMC pyroptosis in AS progression is relatively neglected; (2) although much research has identified novel regulatory mechanisms in pyroptosis of plaque cells, whether they are involved in atheroma formation lacks direct evidence in vivo; (3) considering the involvement of multiple cell types in AS progression, the experiments designed in a single cell type cannot represent the disease in vivo; (4) relevant clinical data are still deficient. We inferred that compared to hard-to-obtain plaque samples, evaluating the levels of pyroptosis-related factors in leukocytes in the peripheral blood of patients may be an alternative scheme; (5) activation of the canonical inflammasome pathway is mainly responsible for pyroptosis-induced inflammation within plaques, whereas the role of the caspase-4/5/11-mediated non-canonical way in AS progression is rarely reported; (6) as gasdermins are critical execution proteins in the pyroptosis process, researchers should investigate the role of gasdermins in the pyroptosis of plaque cells during atherogenesis deeply. Addressing these limitations will undoubtedly deepen our understanding of the role of pyroptosis in AS progression and promote the development of pyroptosis-targeted drugs.

## Data Availability

Not applicable.
